# Supplementary material for: Exploiting Machine Learning Technologies to Study the Compound Effects of Serum Creatinine and Electrolytes on the Risk of Acute Kidney Injury in Intensive Care Units
Source: Diagnostics (Basel). 2023 Jul 31;13(15):2551. doi: 10.3390/diagnostics13152551 (PMC10417601; doi:10.3390/diagnostics13152551)
Supplement: Supplementary file 1 [file diagnostics-13-02551-s001.zip › Supplementary Table S1.pdf]

Supplementary Table S1 The ICD-9 and ICD-10 codes employed to identify post-renal AKI cases.

ICD-9	Codes
Benign prostatic hyperplasia	600.00, 600.01, 600.20, 600.21, 600.90, 600.91, 752.89
Calculus of kidney and urinary tract	592.0, 592.1, 592.9, 593.4, 594.1, 594.8, 594.9, 753.20, 753.3
Urinary Obstruction	599.60, 599.69, 753.6
Benign neoplasm of prostate	222.2
Malignant neoplasm of prostate	185
ICD-10	Code
Obstructive and reflux uropathy	N13.0, N13.1, N13.2, N13.3, N13.4, N13.5, N13.6, N13.7, N13.8, N13.9
Calculus of kidney and ureter	N20.0, N20.1, N20.2, N20.9
Calculus of lower urinary tract	N21.0, N21.1, N21.8, N21.9
Calculus of urinary tract in diseases classified elsewhere	N22
Benign prostatic hyperplasia	N40.0, N40.1, N40.2, N40.3
Malignant neoplasm of prostate	C61
Benign neoplasm of prostate	D29.1
