# Supplementary material for: Exploiting Machine Learning Technologies to Study the Compound Effects of Serum Creatinine and Electrolytes on the Risk of Acute Kidney Injury in Intensive Care Units
Source: Diagnostics (Basel). 2023 Jul 31;13(15):2551. doi: 10.3390/diagnostics13152551 (PMC10417601; doi:10.3390/diagnostics13152551)
Supplement: Supplementary file 1 [file diagnostics-13-02551-s001.zip › Supplementary Table S2.pdf]

Supplementary Table S2 Summary of the software packages employed and parameter ranges.

Model	Package (version)	Parameter ranges
Decision tree	rpart (4.1.19)<br>R (4.2.1)	Method: class
		Cp: 0.01,0.005,0.001
		Prior: 0.4 ~ 0.95 with 0.0005 resolution
		Maxdepth: 5
		Minsplit: 5, 13
Random forest	Xgboost (1.5.2)<br>Python (3.9)	tree_method: gpu_hist
		eta: 0.0001, 0.001, 0.1, 0.3, 0.5, 0.6, 0.8
		max_depth: 3, 5, 6, 7
		min_child_weight: 10, 3, 1, 0.9, 0.5, 0.1
		max_delta_step: 0, 0.3, 0.5, 0.8, 1, 5
		n_estimators: 10, 50, 100, 300
		scale_pos_weight: 0.1, 0.5, 0.7, 1.0, 2, 4, 5, 10, 15, 20, 25, 50, 80, 100
		eval_metric: auc
