# Supplementary material for: Exploiting Machine Learning Technologies to Study the Compound Effects of Serum Creatinine and Electrolytes on the Risk of Acute Kidney Injury in Intensive Care Units
Source: Diagnostics (Basel). 2023 Jul 31;13(15):2551. doi: 10.3390/diagnostics13152551 (PMC10417601; doi:10.3390/diagnostics13152551)
Supplement: Supplementary file 1 [file diagnostics-13-02551-s001.zip › Supplementary Table S3.pdf]

Supplementary Table S3 The detailed performance data observed in the 5-fold cross validation process.

Performance metric	Sensitivity level 0.80			Sensitivity level 0.95		
	Decision tree model	Logistic regression model	Random forest model	Decision tree model	Logistic regression model	Random forest model
Sensitivity	0.798	0.799	0.799	0.949	0.949	0.949
Specificity	0.721	0.773	0.732	0.479	0.414	0.382
Positive predictive value (PPV)	0.116	0.137	0.119	0.076	0.068	0.065
Negative predictive value (NPV)	0.987	0.988	0.988	0.995	0.994	0.994
Accuracy	0.724	0.774	0.735	0.499	0.437	0.407
F1-Measure	0.202	0.235	0.207	0.142	0.127	0.121
Matthews Correlation Coefficient (MCC)	0.232	0.568	0.238	0.175	0..151	0.140
Relative risk	9.845	11.98	10.14	16.89	13.87	13.01
Area Under the Curve (AUC)	0.823	0.857	0.766	0.767	0.854	0.666

True positive (TP): The number of cases that the model predicted to be positive and were truly positive.

True negative (TN): The number of cases that the model predicted to be negative and were truly negative.

False positive (FP): The number of cases that the model predicted to be positive and were reality are negative.

False negative (FN): The number of cases that the model predicted to be negative and were reality are positive.

Sensitivity =$\frac{TP}{TP+FN}$

Specificity =$\frac{TN}{TN+FP}$

PPV =$\frac{TP}{TP+FP}$

NPV =$\frac{TN}{TN+FN}$

Accuracy =$\frac{TP+TN}{TP+TN+FP+FN}$

F1 =$\frac{2\times TP}{2\times TP+FP+FN}$

MCC =$\frac{TP\times TN-FP\times FN}{\sqrt{(TP+FP)\times(TP+FN)\times(TN+FP)\times(TN+FP)}}$

Relative risk =$\frac{TP/(TP+FP)}{FN/(FN+TN)}$
